# Metformin ameliorates valve interstitial cell calcification by promoting autophagic flux

**DOI:** 10.1038/s41598-023-47774-6

**Published:** 2023-12-05

**Authors:** K. Phadwal, X. Tan, E. Koo, D. Zhu, V. E. MacRae

**Affiliations:** 1 The Roslin Institute & R(D)SVS, University of Edinburgh, Easter Bush, Midlothian, EH25 9RG UK; 2 Guangzhou Institute of Cardiovascular Diseases, Key Laboratory of Cardiovascular Diseases, School of Basic Medical Sciences, The Second Affiliated Hospital, Guangzhou Medical University, Guangzhou, 511436 China; 3 Guangzhou Institute of Cardiovascular Diseases, Guangdong Key Laboratory of Vascular Diseases, State Key Laboratory of Respiratory Disease, The Second Affiliated Hospital, School of Basic Medical Sciences, Guangzhou Medical University, Guangzhou, China

**Keywords:** Cell biology, Molecular biology

## Abstract

Calcific aortic valve disease (CAVD) is the most common heart disease of the developed world. It has previously been established that metformin administration reduces arterial calcification via autophagy; however, whether metformin directly regulates CAVD has yet to be elucidated. In the present study we investigated whether metformin alleviates valvular calcification through the autophagy-mediated recycling of Runx2. Calcification was reduced in rat valve interstitial cells (RVICs) by metformin treatment (0.5–1.5 mM) (*P* < 0.01), with a marked decrease in Runx2 protein expression compared to control cells (*P* < 0.05). Additionally, upregulated expression of Atg3 and Atg7 (key proteins required for autophagosome formation), was observed following metformin treatment (1 mM). Blocking autophagic flux using Bafilomycin-A1 revealed colocalisation of Runx2 with LC3 puncta in metformin treated RVICs (*P* < 0.001). Comparable Runx2 accumulation was seen in LC3 positive autolysosomes present within cells that had been treated with both metformin and hydroxychloroquine in combination (*P* < 0.001). Mechanistic studies employing three-way co-immunoprecipitation with Runx2, p62 and LC3 suggested that Runx2 binds to LC3-II upon metformin treatment in VICs. Together these studies suggest that the utilisation of metformin may represent a novel strategy for the treatment of CAVD.

## Introduction

Calcific aortic valve disease (CAVD) is an important global health issue throughout the developed world. It is a chronic disease denoted by progressive fibrotic and calcific valve thickening with reduced ventricular function, which results in left ventricular outflow obstruction^1^. Currently the established therapeutic strategies for CAVD patients are surgical valve replacement or transcatheter aortic valve implantation with prosthetic valves^2^. However, these treatments are invasive, expensive and of high risk for patients, and can result in complications including infection and myocardial infarction. In addition, prosthetic valves have limited longevity, and undergo structural breakdown and calcification^3^. The discovery of new treatment approaches to inhibit the development of CAVD is therefore critical.

The pathophysiology of CAVD shares many similarities to that of physiological bone mineralization^4^. Valve interstitial cells (VICs), the predominant cell type within the aortic heart valve, have a crucial function in CAVD advancement. An extensive number of studies have revealed that VICs are able to undergo osteogenic ‐differentiation and calcification to a bone-like phenotype^5, 6^, with an associated upregulation in the osteogenic marker Runx2^7^. Indeed, targeted ablation of Runx2 has been shown to decrease expression of its osteogenic targets and reduce calcification^8–10^.

Metformin is the most common first-line medication for the treatment of type 2 diabetes (T2D). It helps to restore the body's response to insulin by decreasing the amount of blood sugar that the liver produces, and has been clinically used for over 60 years^11, 12^ . Additionally, metformin can successfully treat patients with T2D with few adverse side-effects^13^. The mechanism underpinning the anti-diabetic action of metformin involves the upregulation of hepatic adenosine monophosphate-activated protein kinase (AMPK) activity, subsequently leading to reduced gluconeogenesis and lipogenesis^14^. However, beneficial effects have also been recently observed in various diseases including cancer^15^, liver disease^16^, obesity^17^ and osteoporosis^18^.

Metformin has also been reported to exert novel cardio-protective actions. Cardiovascular disease incidence is decreased in T2D patients treated with metformin^19^ and cardiac remodelling is also reduced^20, 21^. Furthermore, research has recently demonstrated that metformin induces direct beneficial effects on aortic valve function via the modulation of VIC calcification. Metformin reduces the osteoblastic transdifferentiation of human aortic VICs^22^, and alleviates aortic VIC calcification via activation of the phosphoinositide-3-kinase–/Akt (PI3K/Akt) pathway through a mechanism dependent on AMPK^23^. Concomitant studies have highlighted that the employment of metformin may signify an innovative pharmacological approach against arterial calcification via the stimulation of AMPK-dependent autophagy^24^. A recent seminal observational study by Morciano and colleagues^25^, performed in ex vivo human samples, has further revealed that enhancing autophagy with rapamycin (a potent inducer of autophagy) regresses the calcification phenotype by reducing the apoptosis associated with calcific aortic valve stenosis. However, whether metformin directly modulates autophagic flux in VICs and influences the regression of osteogenic factors has yet to be established.

## Materials and methods

### VIC culture and calcification

The RVIC Sc40T cell line (rat VIC-derived) was established by Capital Biosciences (Gaithersburg, Maryland, USA) as previously described^26^. Cells were seeded in growth media (complete DMEM media, Invitrogen, supplemented with 10% fetal bovine serum and 1% Gentamicin) in 12 well plates at a density of 1 × 10^5^ cells/cm^2^. Calcification was induced as reported previously^7^. Cells were grown to 80% confluence (Day 0), before treating with calcification medium containing 2.7 mM calcium (Ca) and 2.5 mM phosphate (Pi). CaCl_2_ and Na_2_HPO_4_/NaH_2_PO_4_ (Sigma-Aldrich, Dorset, UK) were used to supplement ionic calcium and phosphate in the media^7^. Cells were incubated for up to 3 days in a humidified atmosphere of 95% air/5% CO_2_, and the medium was changed every second/third day. Metformin (Sigma) was added at day 0^26^.

### Determination of calcification

Calcium deposition was quantified by HCl leaching, as previously described by our laboratory^27^. Briefly, cells were washed twice with phosphate buffered saline (PBS) and decalcified for 24 h with 0.6 N HCl at 4 °C. Free calcium was determined calorimetrically by a stable interaction with O-Cresolphthalein using a commercially available kit (Randox Laboratories Ltd., County Antrim, UK) and corrected for total protein concentration (Bio-Rad Laboratories Ltd., Hemel Hempstead, UK)^27^. Absorbances were measured using a Synergy HT microplate reader (BioTek, Swindon, UK) at 570 nm (calcium) and at 690 nm (protein). Calcium deposition was also evaluated by alizarin red staining^28^. Cells were washed twice with PBS, fixed in 10% neutral buffered formalin (NBF) for 15 min, stained with 2% alizarin red (pH 4.2) for 5 min at room temperature (RT) and rinsed with distilled water^28^.

### Autophagic flux inhibition

Autophagic flux was inhibited by treating RVICs with Bafilomycin-A1 (Baf-A, 5nM) and Hydroxychloroquine (HCQ, 10μM) for 72 h. The diluent for Bafilomycin-A1 was dimethyl sulfoxide (DMSO, final concentration 0.3%), with all control cells receiving 0.3% DMSO. The diluent for Hydroxychloroquine was water, with all control cells receiving matching volumes of water.

### Cell viability assay

The alamar blue assay (Invitrogen, DAL1025) was performed according to the manufacturer’s instructions. RVICs treated with metformin and autophagy inhibitors Bafilomycin-A1 (Baf-A, 5 nM) and Hydroxychloroquine (HCQ, 10 μM) for 72 h did not show a significant decline in cell viability compared to untreated cells (Supplementary Fig. 1).

### Cell imaging

Cells were seeded on glass cover slips in 24-well plates at a density of 3 × 10^6^ cells/well. Cells were then fixed with 4% paraformaldehyde (PFA) at 4 °C and washed with PBS^24^. The fixed cells were permeabilised with 0.1% triton X100 (Sigma) and blocked with 5% goat serum prior to incubation with primary antibodies LC3 (1:300; rabbit polyclonal, PM036; MBL International) and Runx2 (1:300; mouse polyclonal, sc‑390,351, Santa Cruz) overnight at 4  °C. After washing cells were incubated for 1 h in the dark with Alexa Fluor@488 anti-rabbit antibody (A11034; Life Technologies) and Alexa Fluor 647 anti-mouse antibody (A21236; Life Technologies)^24^. Cells were then washed with PBS and stained with Hoechst (1:10,000; Sigma). The coverslips were mounted onto slides with Prolong®Gold Anti-Fade Reagent containing DAPI (Life Technologies). Fluorescence signal was detected using a Zeiss LSM 710 inverted confocal microscope (Oberkochen, Germany). ImageJ (WI, USA) was employed to assess the number of LC3 puncta within the cytoplasm^24^.

### Immunofluorescence

Cells were seeded on glass cover slips in 12 well plates at a density of 3 × 10^6^ cells/well. Cells were fixed in 10% neutral buffered formalin (NBF) for 20 min and washed with PBS. The fixed cells were permeabilised with 0.1% triton for 10 min at 4 °C and washed with PBS. Non-specific antibody binding was blocked with 5% goat serum incubation for 1 h. Fixed and permeabilised cells were then incubated with primary antibodies LC3 (1:300; rabbit polyclonal; PM036; MBL International) or a combination of LC3 and Runx2 (1:100; Mouse; Sc-390351; Santa Cruz Biotechnology) overnight at 4 °C^24^. They were washed with PBS and incubated for 1 h in the dark with Alexa Fluor@488 anti-rabbit antibody (A11034; Life Technologies) and Alexa Fluor@647 goat anti mouse antibody (A21236; Life Technologies). Cells were washed in PBS and stained with Hoechst (1:10,000; Sigma) and then mounted onto slides with Prolong®Gold Anti-Fade Reagent (Life Technologies)^24^. Fluorescence signal was observed using a Zeiss LSM 710 inverted confocal microscope (Oberkochen, Germany).

### Western blotting

Cells were lysed in radio-immunoprecipitation assay (RIPA) buffer containing Protease Inhibitor Cocktail (Thermo Fisher Scientific, Waltham, MA, USA) and total protein concentration measured with BCA assay (Thermo Fisher Scientific). Western blotting was undertaken as previously reported by our laboratory^29^. PVDF membranes were probed overnight at 4 °C with primary antibodies (1:1000 dilution in LICOR blocking buffer or 5% milk in TBST), LC3 (1:3000 PM036; MBL International), Atg3 (1:1000, ab108251; Abcam), Runx2 (1:1000, ab236639; Abcam), Bsp (DF7738; Affinity Biosciences), p62 (ab240635; Abcam), Beclin 1 (3495; Cell Signaling Technology) and Atg7 (8558; Cell Signaling Technology). Blots were next incubated in HRP conjugated goat anti-rabbit IgG (P0448; Dako) for 1 h^24^ and subsequently imaged using the GeneGenome system (Syngene, Maryland, USA). Membranes were then washed and re-probed for β-actin expression (BA3R; Invitrogen).

### Co-immunoprecipitation studies

Cells were lysed in Lysis buffer (Cell Signaling Technology) supplemented with Halt™ Protease Inhibitor Cocktail (Thermo Fisher Scientific) and total protein concentration measured with BCA assay (Thermo Fisher Scientific)^7^. Cell lysates (200 μg) were incubated for 12 h at 4 °C either with 5 µg/ml anti-Runx2 (ab236639; Abcam), 5 µg/ml anti-p62 (ab240635; Abcam) or 5 µg/ml anti-LC3 (PM036; MBL International) or 5 µg/ml anti-rabbit IgG (7074; Cell Signaling Technology^24^. Next, lysates were incubated with 20 µl Protein G magnetic agarose beads (73,778; Cell Signaling Technology) for 30 min at RT. The beads were then washed with lysis buffer, pelleted using a magnetic rack and boiled in NuPAGE LDS sample buffer with NuPAGE sample reducing agent (Thermo Fisher Scientific) prior to western blotting analysis with Runx2 (ab236639; Abcam), p62 (ab240635; Abcam) or LC3 (PM036; MBL International) antibodies^24^. 10% of the lysate was re-assessed to determine loading input. Quantification of co-immunoprecipitation (Co-IP) was undertaken using Image J.

### Statistical analysis

All data are presented as mean ± SEM. Data were analyzed by unpaired t test or one-way analysis of variance (ANOVA) followed by Tukey's range test, as appropriate. Statistical analysis was performed using GraphPad prism software (CA, USA). *P* < 0.05 were considered to be significant, and *p* values are represented as *p < 0.05; **p < 0.01; ***p < 0.001; ****p < 0.0001.

## Results

### Metformin alleviates VIC calcification

**Figure 1:**
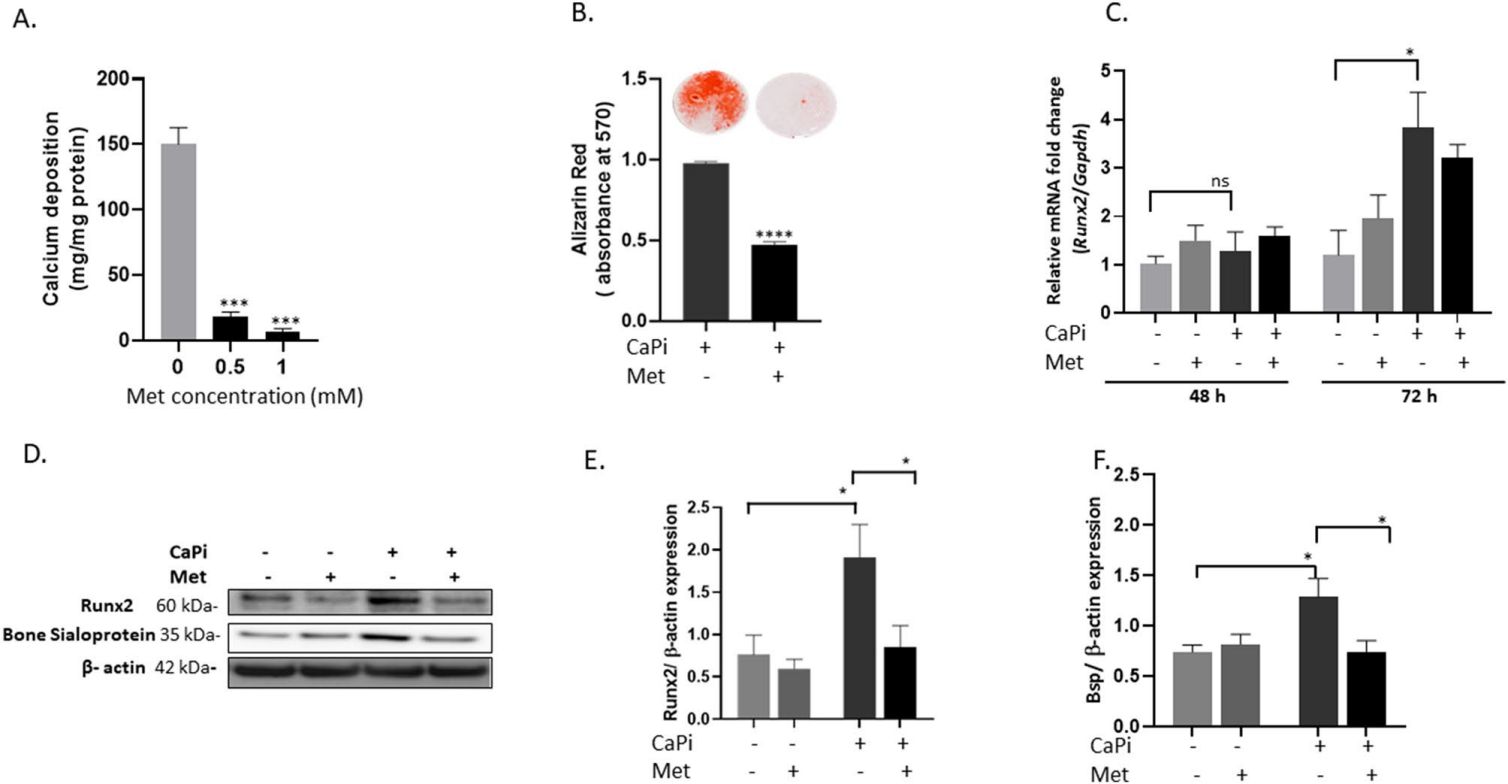
Metformin inhibits calcification in RVICs and reduces the induction of RUNX-2 and bone sialoprotein expression. (**A**) Calcium content (mg/mg protein) of cells treated with metformin (Met) (0.5–1.0 mM), (n = 3). (**B**) Alizarin red staining and its quantification in cells treated with metformin (1 mM), (n = 3). (**C**) mRNA expression of *Runx2* in calcified RVICs cultured in presence/absence of metformin (1 mM) for 48 and 72 h. (n = 3). (**D**) Representative western blots and (**E**, **F**) western blot quantification showing the effect of metformin treatment (1 mM) on the protein levels of Runx2 and bone sialoprotein compared to β-actin (n = 4). Data shown as mean + / − S.E.M *P < *0.05*; **P < *0.01*; ***P < *0.001* compared to control. Full-length blots are presented in Supplementary Fig. 2.

**Figure 2:**
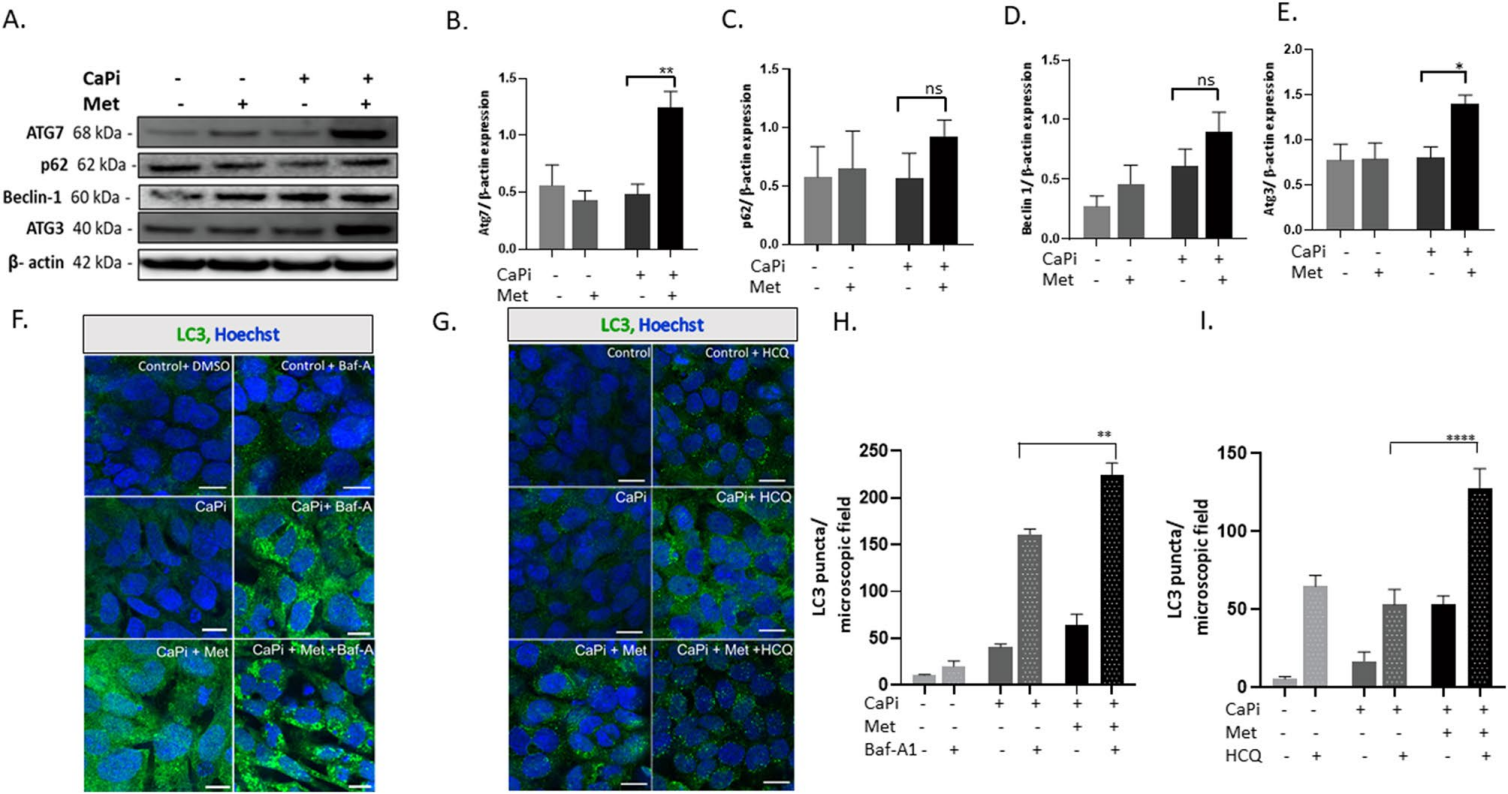
Metformin induces autophagy in calcified RVICs. RVICs were cultured in the presence/absence of calcium (2.5 mM) and phosphate (2.7 mM Pi) with 0.3% DMSO for 72 h in the presence/absence of metformin (Met) (1mM) and/or 5nM Bafilomycin-A1 (Baf-A) or 10 μM Hydroxychloroquine (HCQ). (**A**) Representative western blots and (**B**–**E**) western blot quantification showing the effect of metformin (1 mM) on Atg7, Beclin-1, Atg3 and p62 expression compared with β-actin (n = 4). (**F**, **G**) Representative confocal images showing the effect of Bafilomycin-A (Baf-A; 5 nM) and Hydroxychloroquine (10 μM) and/or metformin treatment on LC3 expression (n = 3; scale bar = 10 μm) with (**H**, **I**) quantification of LC3 puncta. Data shown as mean + / − S.E.M *P < *0.05*, ***P < *0.001*, ****P < *0.0001* compared to control. Full-length blots are presented in Supplementary Fig. 2.

Our initial studies investigated the effect of metformin administration on VIC calcification. Calcification was reduced in VICs cultured in the presence of metformin at both 0.5mM and 1mM concentrations, compared with control cells (Fig. 1A; *P* < 0.001). This was further confirmed with reduced alizarin red staining seen in metformin (1mM) treated RVICs (Fig. 1B; *P* < 0.0001). Next, we examined if metformin could induce changes in the expression levels of Runx2, a critical transcription factor for osteoblastogenesis, under calcifying conditions. Elevated mRNA and protein levels of Runx2 were observed in cells cultured in pro-calcifying medium after 72 h. Interestingly, metformin treatment did not revert this increase in mRNA expression. However, reduced protein expression of Runx2 itself, and it’s downstream target bone sialoprotein (Bsp)^31^ (Fig. 1C–F), were noted at this time point. Additionally, upregulated expression of Atg3 and Atg7 was noted in calcifying VICs cultured in the presence of metformin for 72 h (1mM) (Fig. 2A,B and E, *P* < 0.001, *P* < 0.05). Atg3 and Atg7 catalyse the conjugation of LC3-I with phosphatidylethanolamine to form LC3-II, a key step in autophagosome formation^30, 31^.The expression of Beclin-1 and p62 (Seqestome1) was also assessed; both remained unchanged following 72 h of metformin treatment (Fig. 2A,C and D). Beclin-1 regulates autophagy by initialising the assembly of autophagosomes^32^ and p62 binds to ubiquitinated proteins and binds them to LC3-II for degradation with in the autophagosomes. In summary, our data suggest that metformin treatment reduces the expression of osteogenic regulators with a simultaneous induction of key autophagy markers required for autophagosome formation.

### Metformin induces autophagic degradation of Runx2

We next determined if metformin modulates autophagic flux in calcifying VICs. VICs cultured under calcifying conditions were cultured with Bafilomycin-A1 (Baf-A; 5 nM) and Hydroxychloroquine (HCQ; 10 μM)^29, 33^. Both compounds have been shown to block autophagic flux by inhibiting the fusion of autophagosomes with lysosomes. VICs cultured under calcifying conditions in the presence of metformin (1mM), Baf-A (5 nM) and HCQ (10 μM) demonstrated an increase in LC3 puncta (Fig. 2F,G,H and I, *P* < 0.001) compared to control conditions, suggesting that autophagic flux is induced in the presence of metformin. The differences in the size, shape and number of LC3-II puncta observed following treatment with Baf-A versus HCQ likely reflect differing modes of action. Baf-A impairs autophagic flux by inhibiting lysosomal degradation capacity, whereas HCQ exerts its action by decreasing autophagosome-lysosome fusion^33^. Together, our immunoblotting and immunofluorescence approaches revealed that metformin treatment in combination with these established inhibitors promoted autophagic flux.

**Figure 3:**
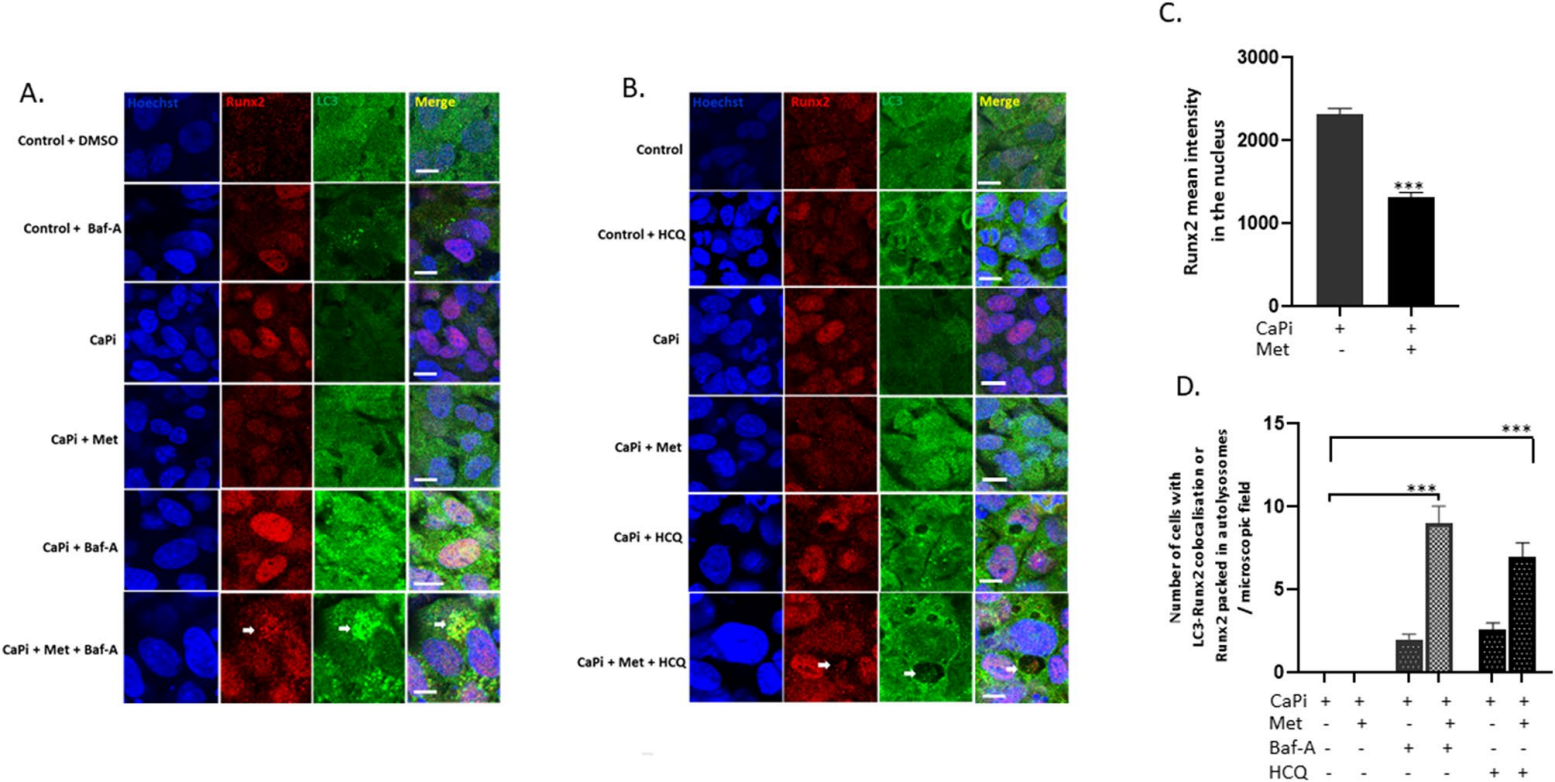
Treatment of calcified RVICs with metformin leads to colocalisation of Runx2 with LC3 in autophagosomes. RVICs were cultured with calcium (2.5 mM) and phosphate (2.7mM Pi) with 0.3% DMSO for 72 h in the presence/absence of 1mM metformin (Met) and/or 5nM Bafilomycin-A1 (Baf-A) or 10μM Hydroxychloroquine (HCQ). (**A**) Representative confocal images showing Runx2 and LC3 staining in Baf-A cells (**B**) Representative confocal images showing Runx2 and LC3 staining in HCQ treated cells. (**C**) Reduced expression of Runx2 in the nucleus of the calcified RVICs treated with metformin (quantified on nuclei from lanes 1 and 2). (**D**) Colocalisation of Runx2 with LC3-II puncta in both Baf-A and HCQ treated cells. Data shown as mean + / − S.E.M ***P < 0.001 compared to control.

**Figure 4:**
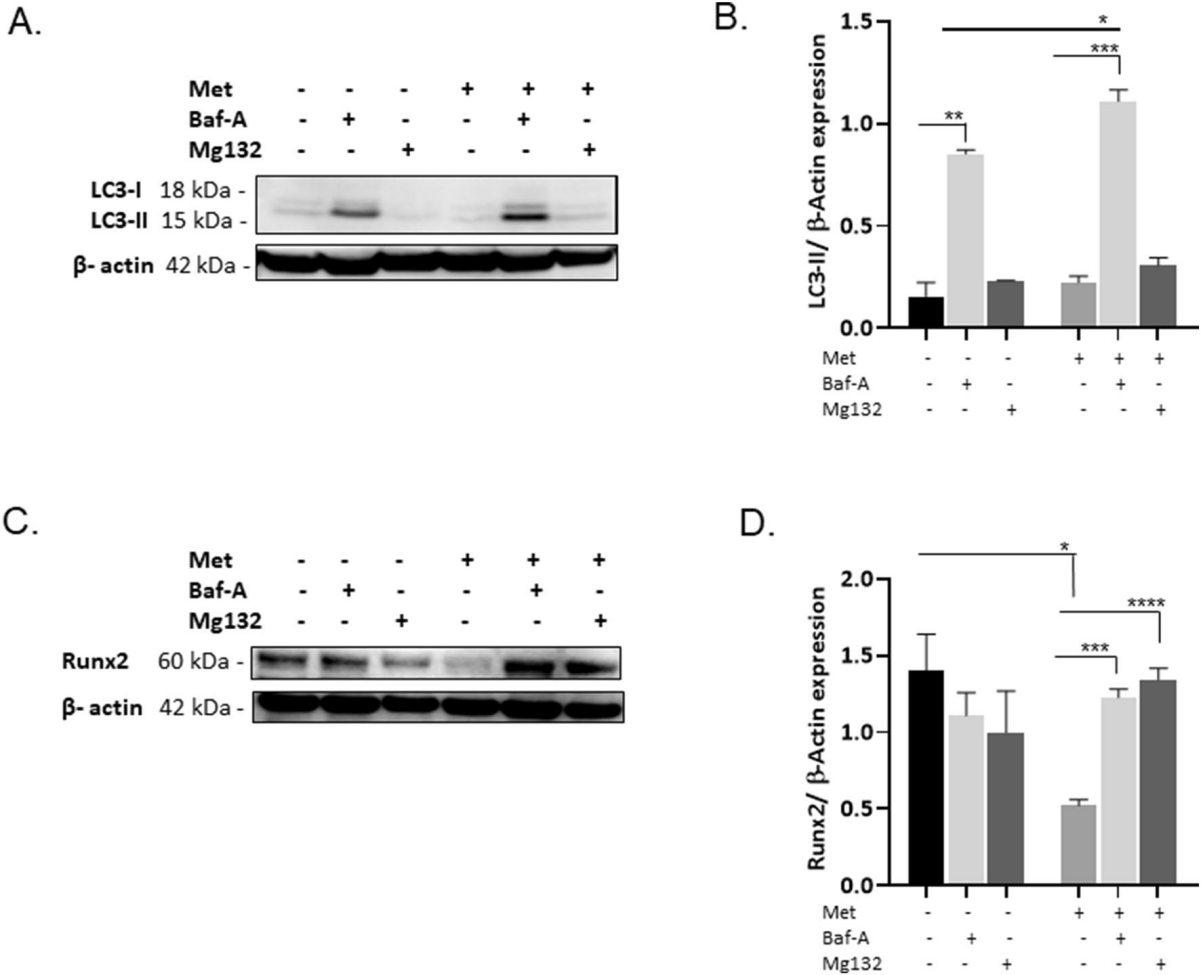
Treatment of calcified VICs with metformin leads to Runx2 degradation via autophagy and the ubiquitin proteasome pathway. RVICs were cultured with calcium (2.5mM) and phosphate (2.7 mM Pi) with 0.3% DMSO for 72 h in the presence/absence of 1mM metformin (Met) and/or 5nM Bafilomycin-A1 (Baf-A) or 50nM MG132. (**A**) Representative western blots and (**B**) western blot quantification showing LC3-II expression compared with β-actin (n = 3), MG132 was used as a negative control (**C**) Representative western blots and (**D**) western blot quantification showing Runx2 expression compared with β-actin (n = 3), MG132 was used to block the ubiquitin proteasome pathway. Data shown as mean + / − S.E.M *P < 0.05; ***P < 0.001; ****P < 0.0001 compared to control. Full-length blots are presented in Supplementary Fig. 2.

Reduced expression of Runx2 within the nuclei of cells treated with metformin was observed (Fig. 3A–C). In addition, colocalisation of LC3 and Runx2 was noted upon addition of Baf-A. Furthermore, the capture of Runx2 within autolysosomes was seen in cells treated with HCQ (Fig. 3A,B and D). A comparable build-up of LC3-II (Fig. 4A and B, *P* < 0.001) and Runx2 protein expression was observed in VICs treated with metformin when autophagic flux was blocked with Baf-A for 72 h (Fig. 4C and D, *P* < 0.001). As a positive control, we utilised the proteasome inhibitor MG132 (50 nM), as the proteasome pathway has been established to play a role in Runx2 degradation^34–36^.

The high autophagic turnover of LC3-II by metformin treatment alone may have resulted in LC3-I to LC3-II conversion passing undetected by immunoblotting. Certainly, a small number of LC3 puncta were noted by immunofluorescence assessment (Fig. 2F and G). However, it is important to highlight that autophagic flux can only be truly assessed with the addition of Baf-A or HCQ^37^.

Together these data suggest that metformin exerts its protective effects against VIC calcification by enhancing autophagic flux, and the subsequent targeting of Runx2 to autophagosomes for degradation.

### Metformin induces LC3-II mediated sequestering of Runx2 in calcified RVICs

We next investigated if metformin was exerting its action through a mechanism involving LC3-II and p62, a selective autophagy receptor which links ubiquitinated cargo with LC3-II for autophagic degradation^38^.

**Figure 5:**
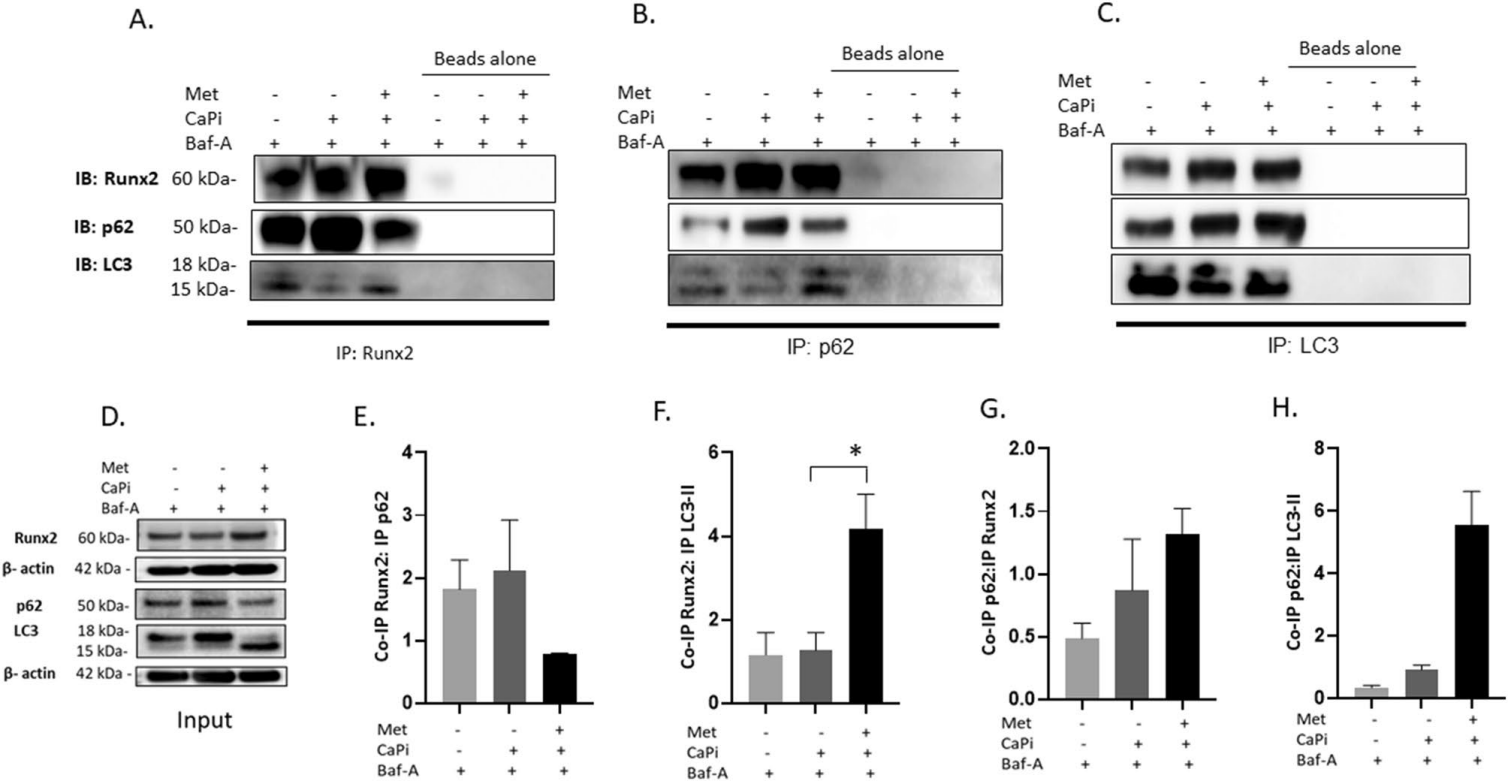
Metformin induces LC3-II mediated sequestering of Runx2 in calcified RVICs autophagosomes/autolysosomes. RVICs were cultured with calcium (2.5 mM) and phosphate (2.7 mM Pi) with 0.3% DMSO for 72 h in the presence/absence of 1 mM metformin (Met) and/or 5nM Bafilomycin-A1 (Baf-A). Representative western blots for Runx2, p62 and LC3 expression with (**A**) Runx2 co-immuno precipitate (IP) lysate (**B**) p62 co-IP lysate and (**C**) LC3 co-IP lysate. Beads alone were used as a negative control (n = 2). (**D**) Input blots for the IP. (**E**–**H**) Quantification of ratios of Co-IP Runx2: IP p62, Co-IP Runx2: IP LC3-II, Co-IP p62: IP Runx2 and Co-IP p62: IP LC3-II. Data shown as mean + / − S.E.M *P < 0.05. Full-length blots are presented in Supplementary Fig. 2.

VICs were treated with Baf-A (5nM) under control versus calcified conditions, in the presence or absence of metformin (1mM). Three-way co-immunoprecipitation (co-IP) of protein lysates with Runx2, p62 and LC3 demonstrated that Runx2 interacts with LC3-II in both control and metformin treated cells (Fig. 5A,B,C,F and H). However, Runx2-LC3-II binding was significantly reduced in calcified VICs cultured in the absence of metformin (Fig. 5A,B, lane 2). Interestingly, reduced p62 protein expression was observed in metformin treated Co-IP lanes (Fig. 5A,B, lane 3). These data may reflect accelerated p62 degradation within the autophagosomes^39^ as a result of metformin-induced autophagic flux. Despite this reduction in expression levels, we propose that the presence of p62 protein within the cells would still permit interaction with both Runx2 and LC3-II in the metformin treated samples (Fig. 5A–C E and G). Reduced p62 expression was also be seen in RVICs treated with metformin and Baf-A in combination (Fig. 5D, input lane 2). Together these data strongly indicate that metformin selectively targets Runx2 for clearance within LC3-II positive autophagosomes and that this interaction may be mediated through the p62 autophagy receptor.

Interestingly p62 levels were decreased following simultaneous treatment with metformin and Baf-A (Fig. 5D, input lane 3). Here, p62 may be serving as an adaptor for both the proteasome and ubiquitin pathways^40^. Indeed metformin has been previously shown to enhance not only ubiquitin mediated proteolysis but also ubiquitin mediated autophagy^41^. In addition, the dynamics of p62 degradation within autophagosomes have been reported to vary between cell lines. Indeed, our findings in RVICs are supported by recent studies in the mouse embryonic fibroblast (MEF) cell line, which also showed reduced p62 expression with concomitant increased LC3-II levels following treatment with Baf-A to block autophagic flux^42^.

**Figure 6:**
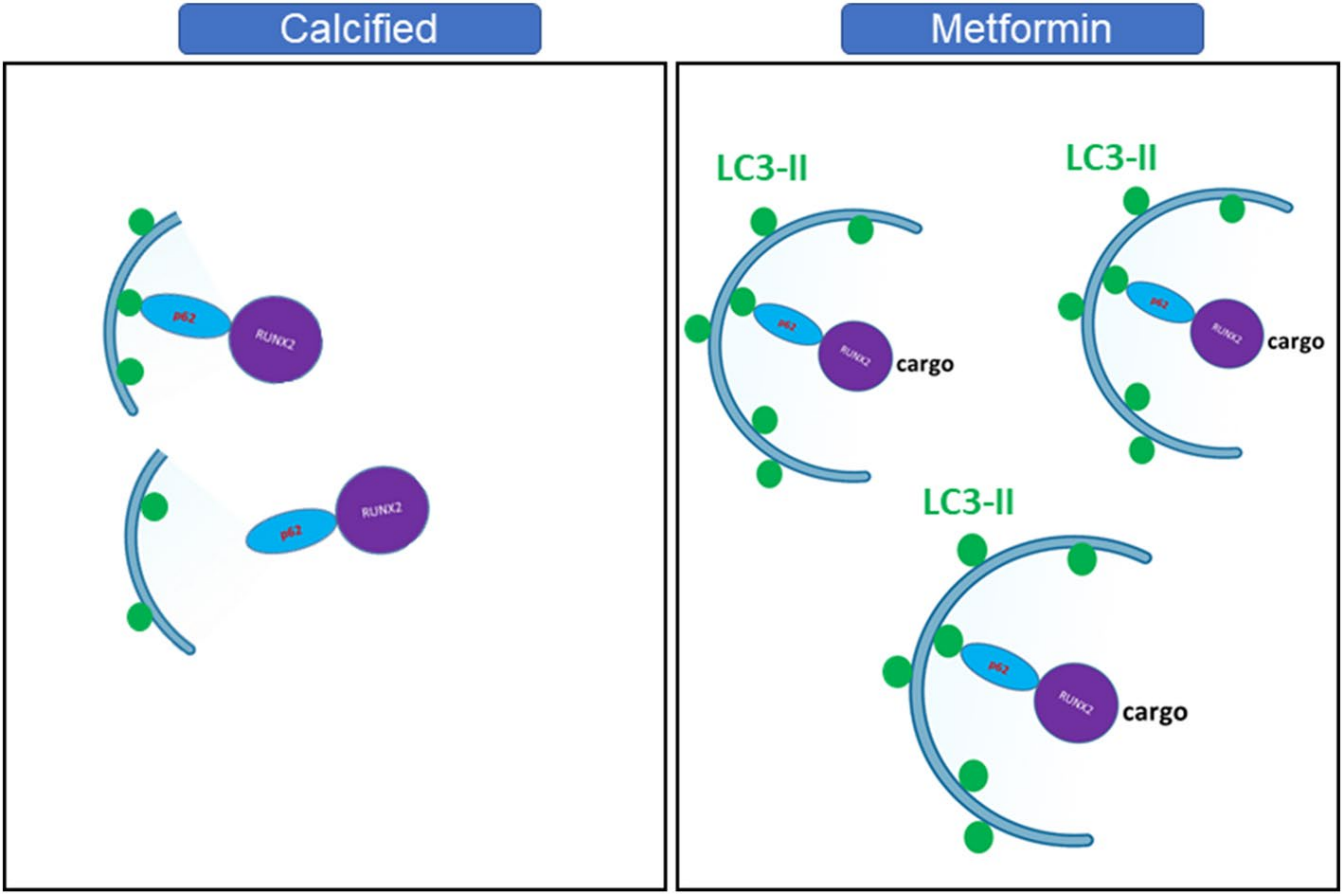
Proposed mechanism of action for metformin in CAVD. Metformin is proposed to decrease calcification in RVICs through the selective binding of Runx2 with mature autophagosome marker LC3-II and the autophagy adaptor p62.

In summary, this study suggests that Runx2 is a cargo for autophagosomes. Our data suggests that treating calcified VICs with metformin enhances autophagy and restores the autophagic breakdown of Runx2 via LC3-II (Fig. 6).

## Discussion

CAVD is the most common valvular heart disease^1^; however, there are currently no effective treatments to impair the progression of this disease. Drug repurposing is therefore an appealing strategy, because it involves the use of de-risked compounds, with potentially reduced development costs and shorter development timelines^43^. With growing evidence suggesting beneficial health benefits beyond its capacity to modulate glucose metabolism^44^, metformin offers an exciting therapeutic option for CAVD.

Metformin is currently the most frequently employed treatment worldwide for type 2 diabetes^45^. However, it is now well established that metformin exerts remarkable changes in cardiovascular function^46^. Metformin has been reported to reduce the occurrence of cardiovascular diseases in T2D patients^19^, with concomitant improved survival and reduced prevalence of cardiac complications in peripheral arterial disease patients^47^. Furthermore, it has been shown to preclude cardiovascular dysfunction in a mouse model of adult congenital heart disease^48^. Recently, metformin has been reported to exert protective effects against vascular calcification, with clinical studies showing that treatment is associated with a reduced below-the-knee arterial calcification score^49^ and decreased progression of atherosclerotic plaques^50^, with in vitro studies demonstrating reduced VSMC calcification^24^. In this study we confirm previous work demonstrating that metformin can effectively inhibit the calcification of VICs^18, 23^, extending these findings to an in vitro animal model of CAVD for the first time.

Recent research has proposed that metformin may exert cardio-protective effects through elevated autophagy^51, 52^. Autophagy is an evolutionarily conserved catabolic process that is crucial for maintaining cellular, tissue and organismal homeostasis. Emerging evidence has demonstrated that autophagy directly protects against cardiovascular calcification by regulating the release of mineralizing matrix vesicles from VSMCs^53, 54^. With recent findings highlighting an important role for VIC-derived MVs in aortic valve calcification^7^, it is possible to speculate that a comparable autophagic mechanism may also underpin CAVD. Indeed a recent translational study has highlighted that the human CAVD phenotype includes defects in autophagy control mechanisms^25^.

The present study is the first to highlight enhanced autophagic flux as the mechanism underpinning the inhibitory effects of metformin on VIC calcification. Metformin-treated VICs showed upregulated LC3II/I expression compared to control cells, in agreement with previous studies in the rat A7r5 thoracic aorta VSMC line^51^ and primary murine VSMCs^24^. Furthermore, we reveal that expression of the autophagy regulator Atg3, Atg7 and LC3 are negatively associated with valve interstitial cell calcification. Atg3 and Atg7 are the key genes involved in autophagy, and acts as E1 and E2 ubiquitin-like conjugating enzymes in the Atg8 conjugation system, contributing to phagophore elongation^55, 56^ by lipidation of LC3-I to LC3-II. These data also support our recent work demonstrating a functional role for Atg3 in the arterial calcification process^24^. Although, a change in expression of p62 at the basal level was not observed between treatments (Fig. 2A), a decrease in p62 expression was seen in metformin and Baf-A treated samples (Fig. 5D) compared to calcified RVICs alone, suggesting increased autophagic degradation following metformin treatment.

Reactive oxygen species (ROS) are an important mediator of autophagy, whereby ROS oxidizes ATG proteins to inhibit autophagy. Interestingly, recent work by En and colleagues has shown that metformin inhibits the ROS-mediated oxidative stress of VICs cultured under calcifying conditions^23^. Analysis of ex vivo human samples has revealed that impairment of mitophagy accompanies CAVD^25^, with parallel studies from our laboratory showing that arterial calcification is associated with increased mitochondrial ROS production and reduced mitophagy (the removal of damaged mitochondria through autophagy)^57^. These data together expose the need for future investigative efforts into the impact of metformin treatment on mitochondrial function and mitophagy in CAVD.

Our investigations have revealed that the treatment of calcifying VICs with metformin reduces the expression of Runx2, a recognised osteogenic transcription factor. These data support previous work demonstrating that metformin effectively ameliorates the osteoblastic differentiation of human VICs following treatment with TGFβ^22^. We further used a co-immunoprecipitation approach to show that metformin directly reduces Runx2 levels in VICs by facilitating its binding to LC3-II. While studies employing co-immunoprecipitation with p62 suggested that metformin may facilitate the interaction between Runx2 and p62, this was not observed following co-immunoprecipitation with Runx2. Further studies are therefore required to establish the specific role of p62 in the sequestering of Runx2 within autophagosomes. These findings extend recent studies demonstrating a link between autophagy and p62 in VSMC calcification^24, 58^, and are further supported by clinical investigations which show disrupted autophagic activity in calcified aortic valve samples from human patients^25, 46, 59^. Although, Runx2 lacks the traditional LIR motif for binding to LC3-II, future investigation into the presence of an alternative motif enabling binding to LC3-II domain would be of interest^60^. Together our studies suggest that exploitation of metformin and its analogues may represent a novel therapeutic strategy for clinical intervention against CAVD.

### Supplementary Information

Supplementary Figure 1.

Supplementary Figure 2.

## Data Availability

All data generated or analysed during this study are included in this published article [and its supplementary information files].
