# Supplementary figures and images for: Metformin ameliorates valve interstitial cell calcification by promoting autophagic flux
Source: Sci Rep. 2023 Dec 5;13:21435. doi: 10.1038/s41598-023-47774-6 (PMC10698150; doi:10.1038/s41598-023-47774-6)

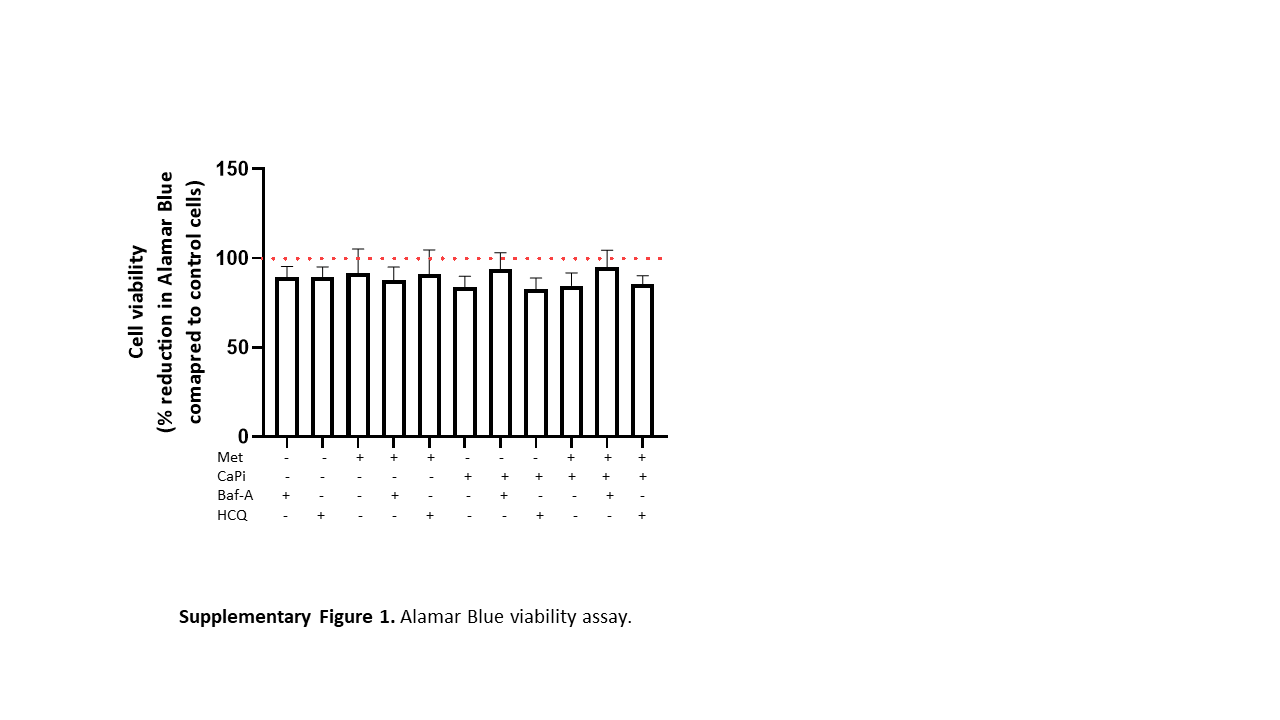

Supplement: Supplementary file 1 — Supplementary Figure 1. [file 41598_2023_47774_MOESM1_ESM.tif]
