# Supplementary material for: Metformin ameliorates valve interstitial cell calcification by promoting autophagic flux
Source: Sci Rep. 2023 Dec 5;13:21435. doi: 10.1038/s41598-023-47774-6 (PMC10698150; doi:10.1038/s41598-023-47774-6)

Supplementary Figure 2: Full length blots

Figure 1D

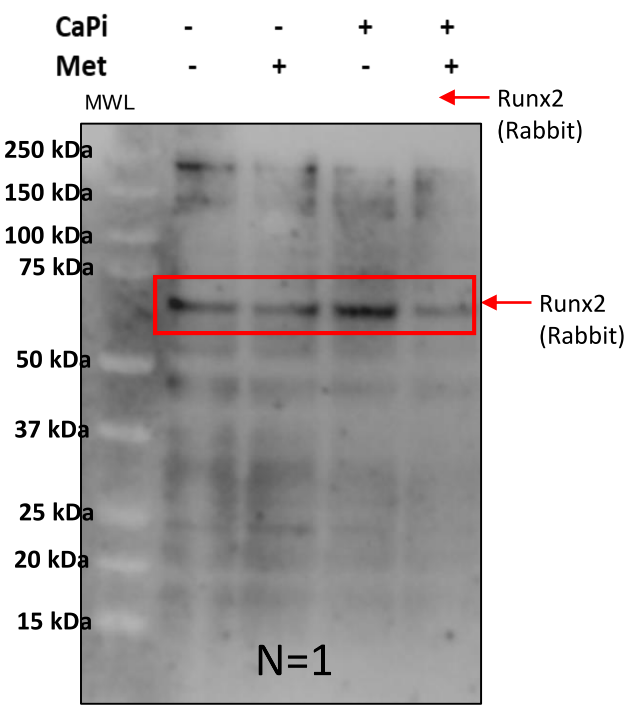

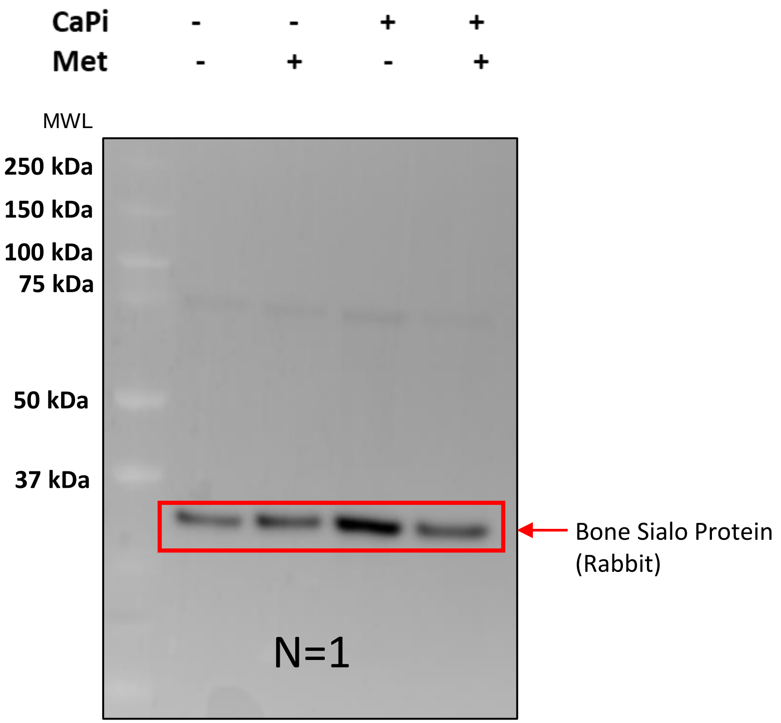

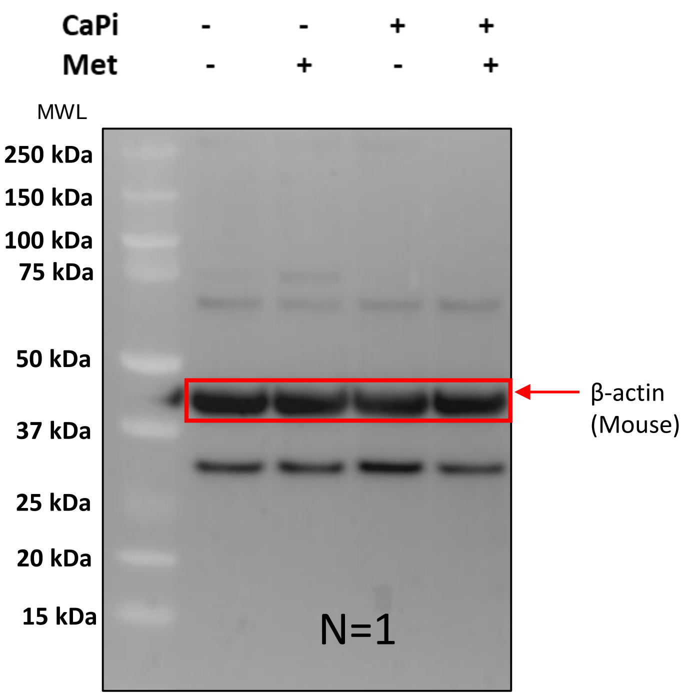

Figure 1D

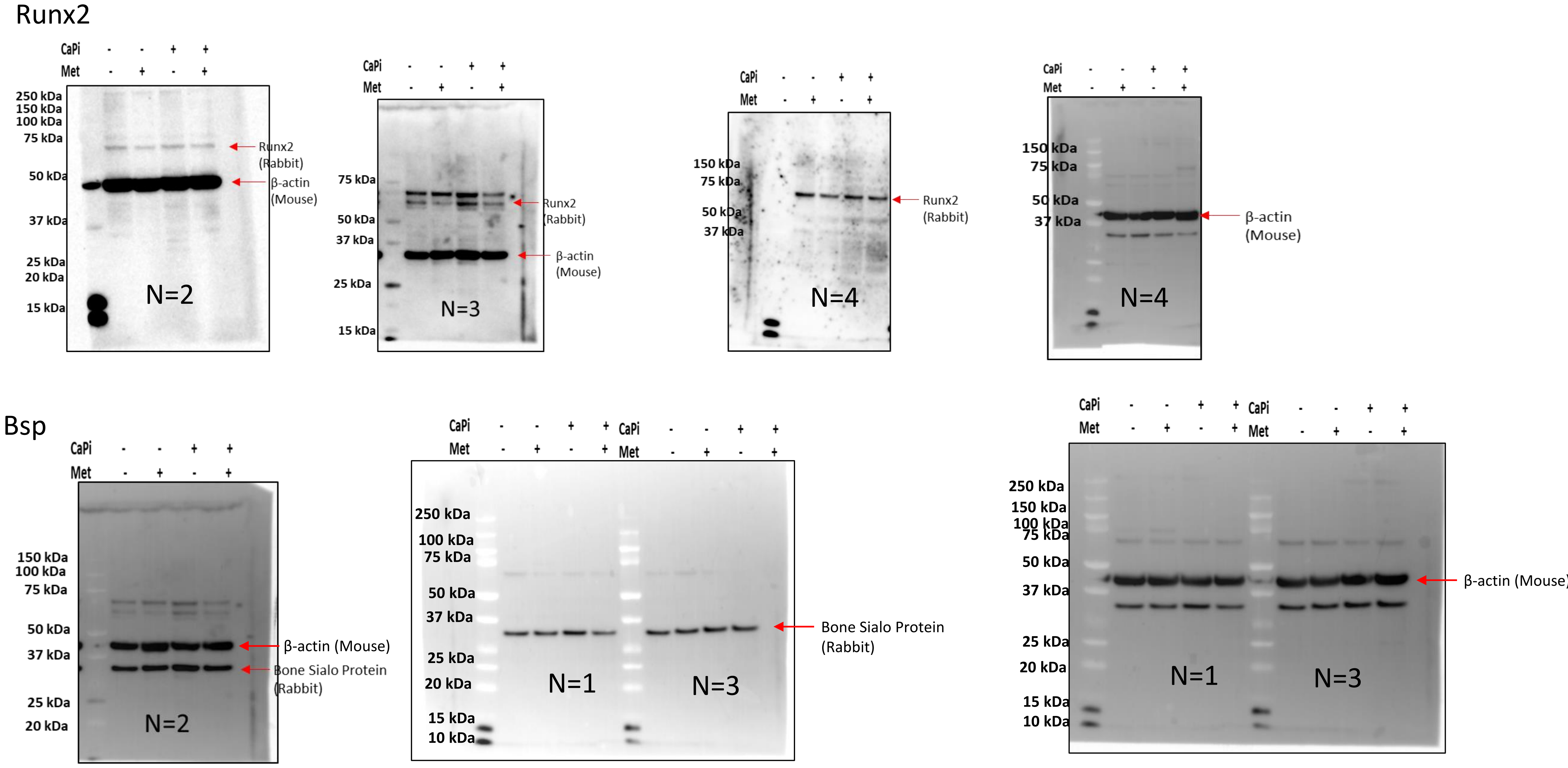
Runx2
CaPi - - + +
Met - + - +
250 kDa
150 kDa
100 kDa
75 kDa
50 kDa
37 kDa
25 kDa
20 kDa
15 kDa
Runx2 (Rabbit)
β-actin (Mouse)
N=2
N=3
N=4
N=4
Bsp
Bone Sialo Protein (Rabbit)
10 kDa
N=1
N=3

Figure 2A

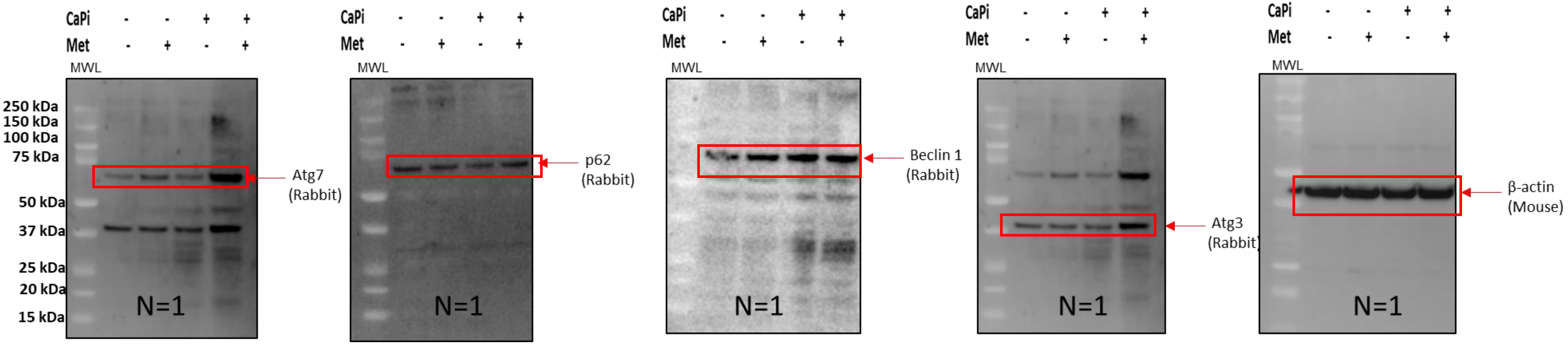

CaPi - - + +
Met - + - +
MWL
250 kDa
150 kDa
100 kDa
75 kDa
50 kDa
37 kDa
25 kDa
20 kDa
15 kDa
N=1
Atg7 (Rabbit)
p62 (Rabbit)
Beclin 1 (Rabbit)
Atg3 (Rabbit)
β-actin (Mouse)

Figure 2A

Atg7

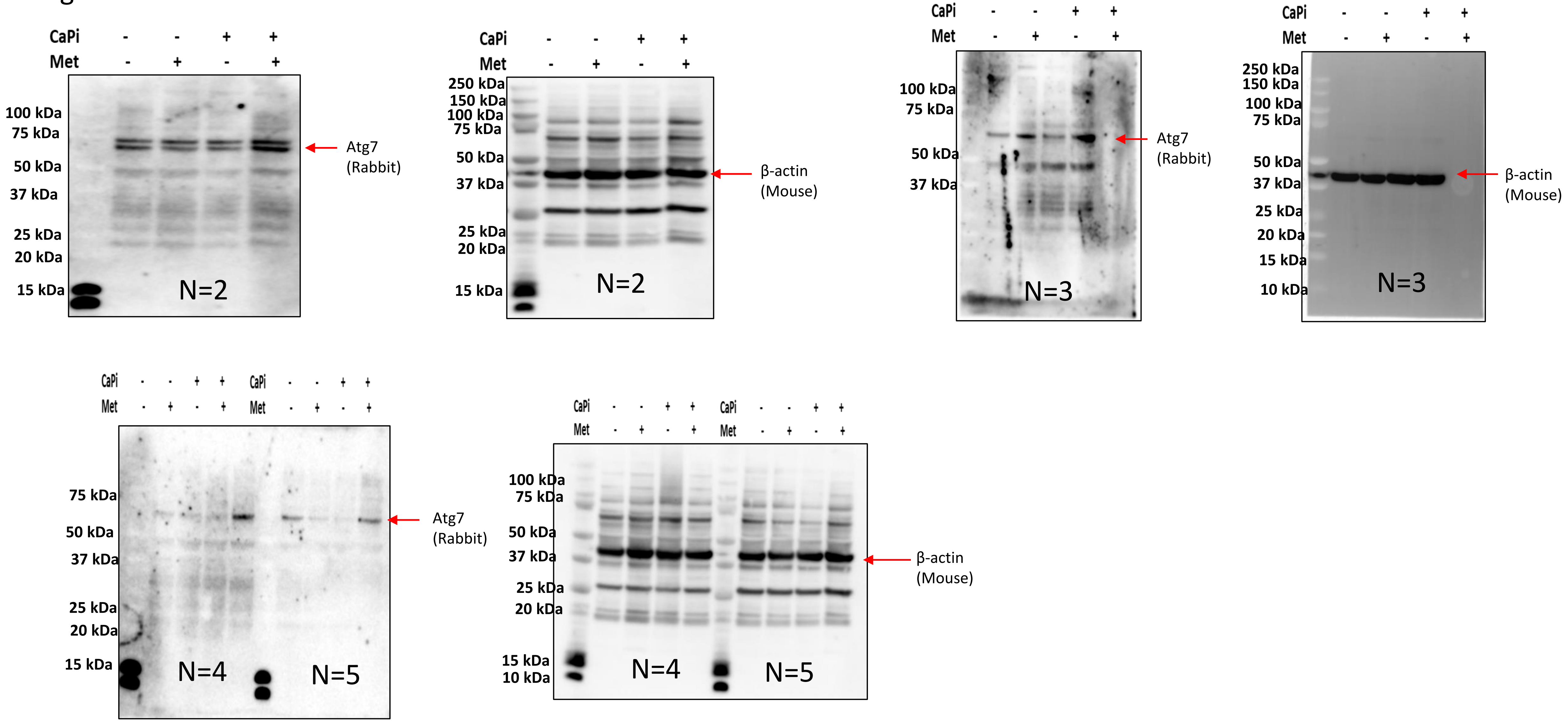

Figure 2A

p62

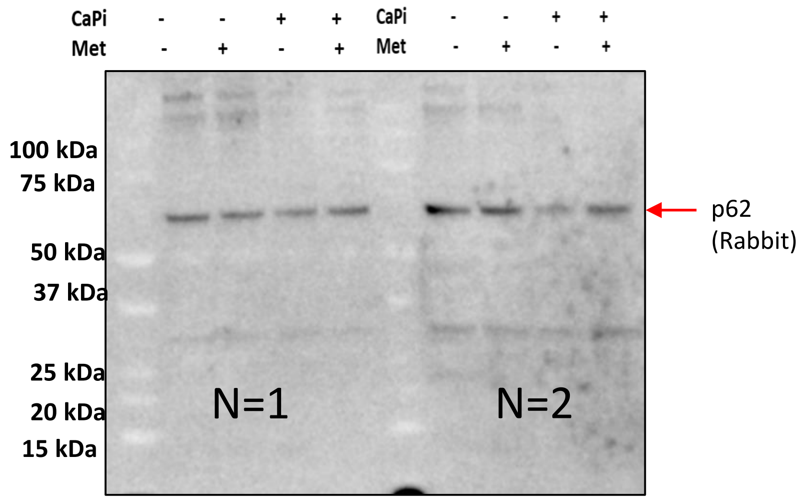

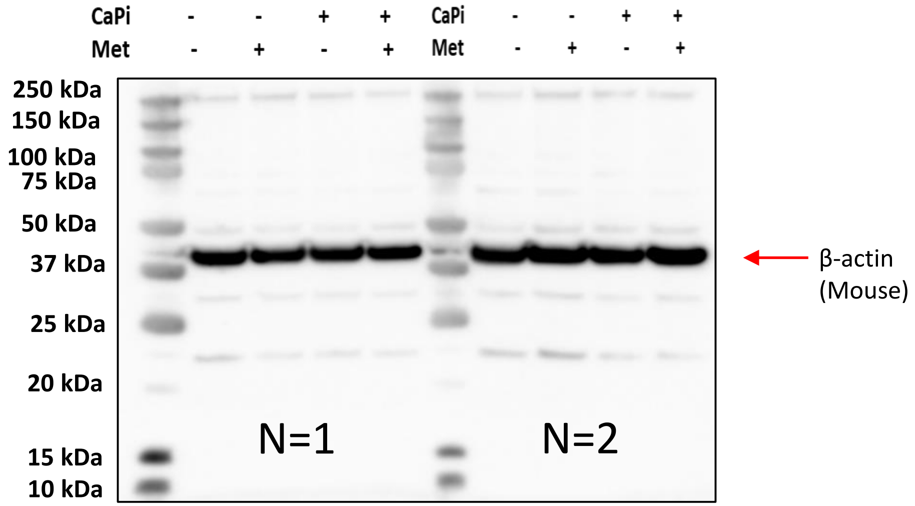

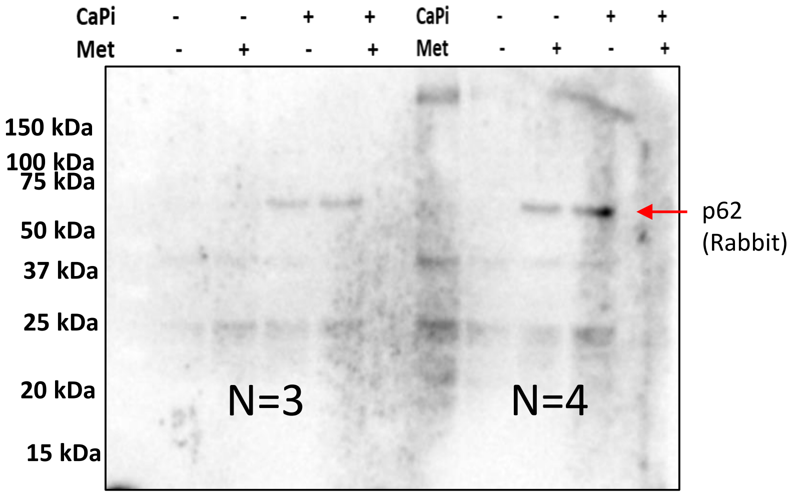

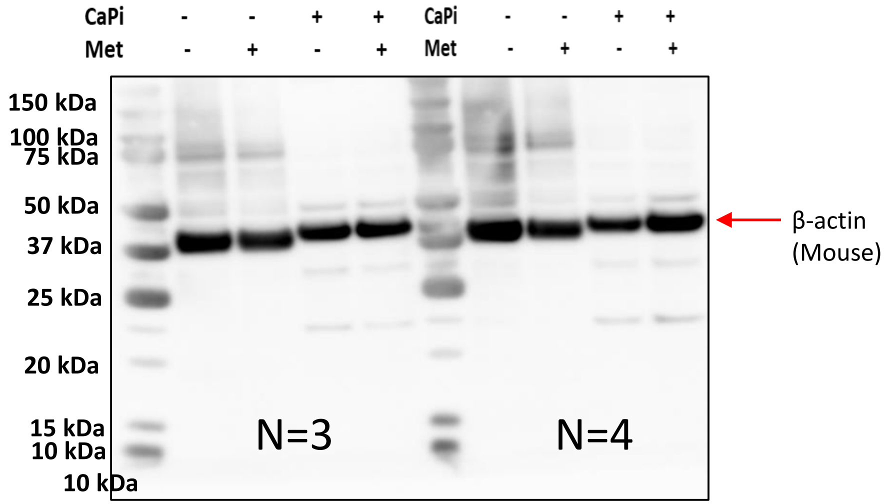

Figure 2A

Atg3

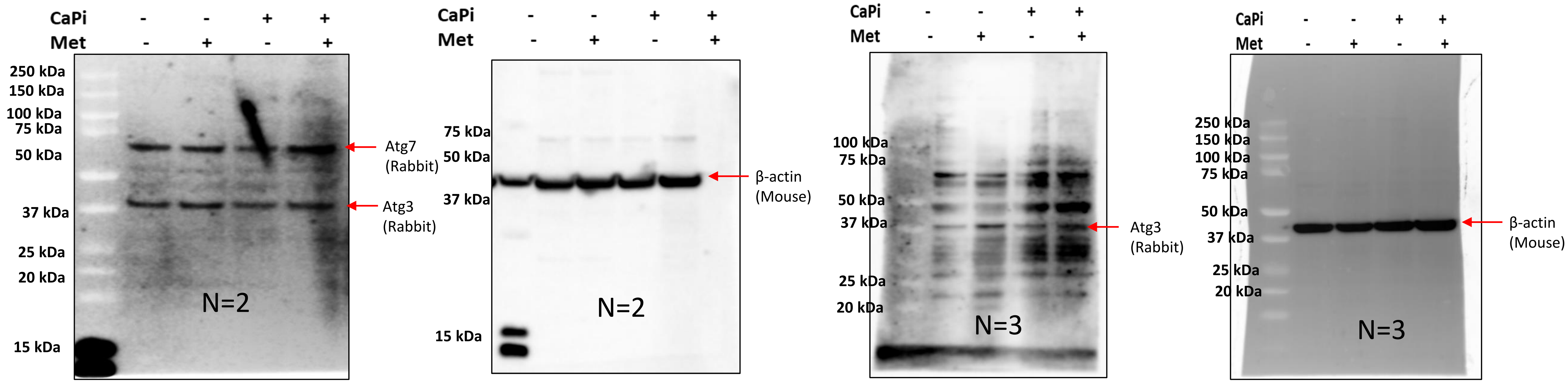

Beclin 1

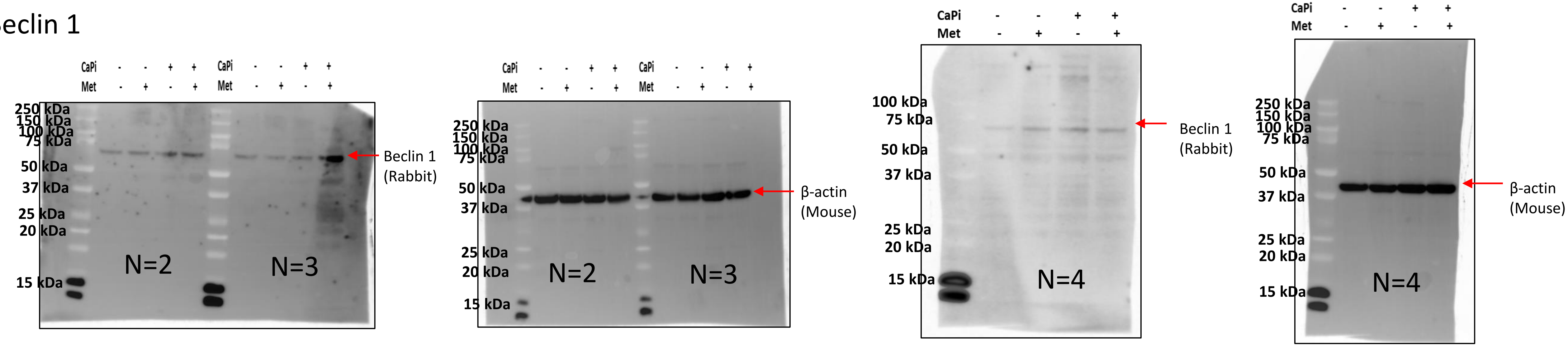

Figure 4A

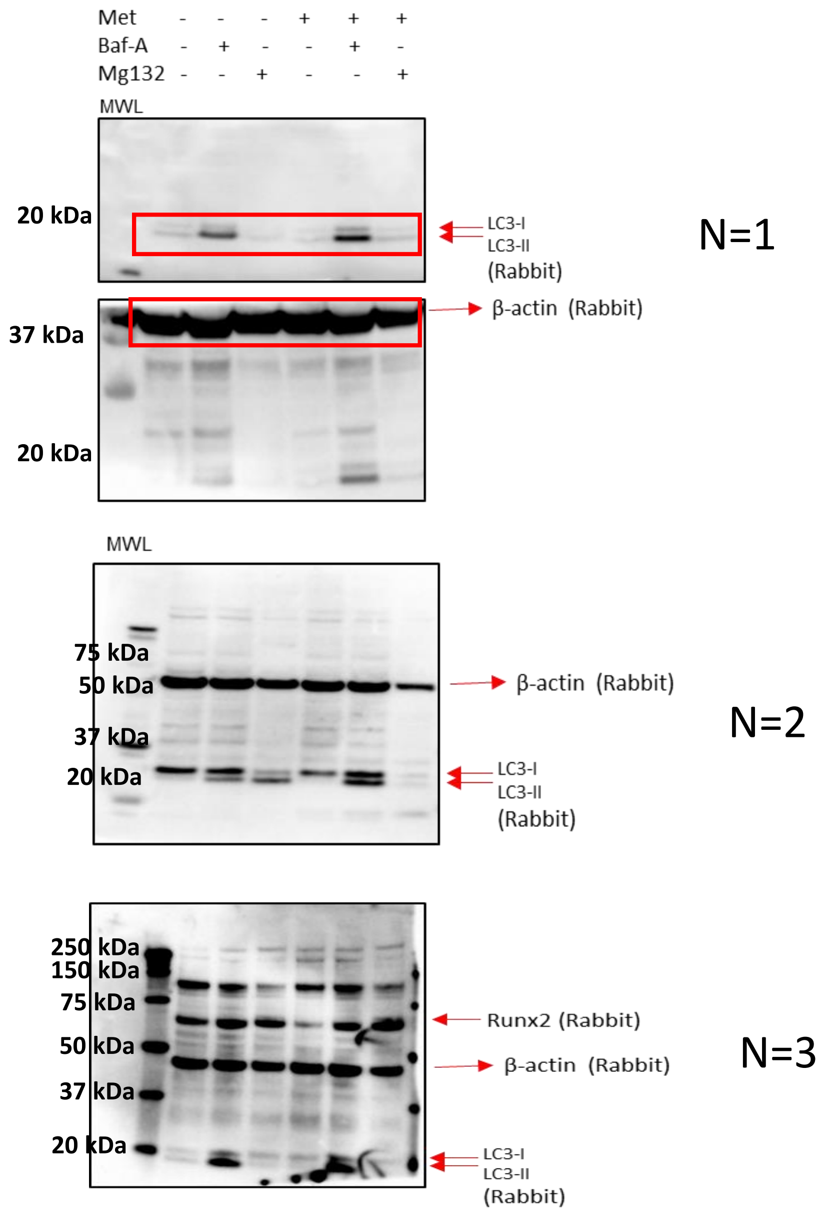

Figure 4C

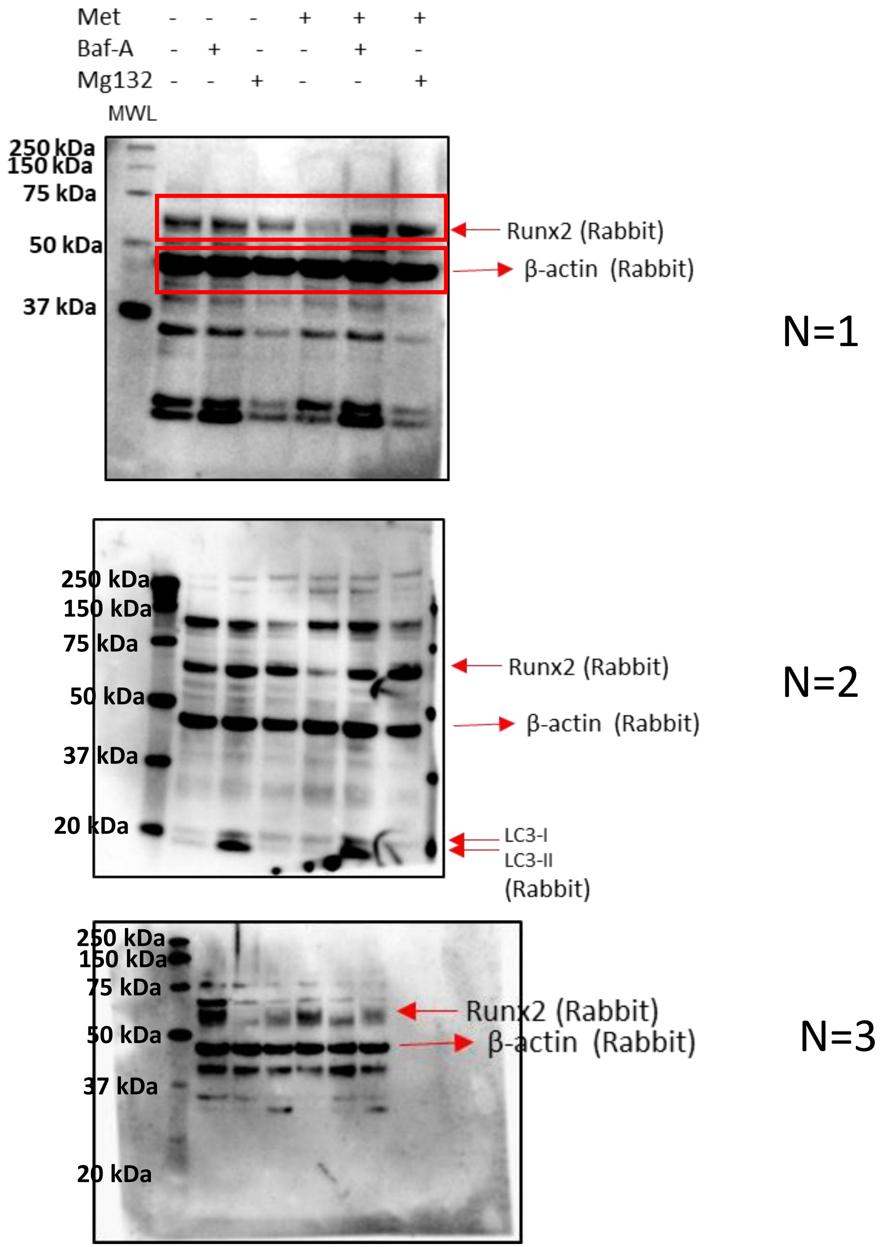

Figure 5A IP RUNX2

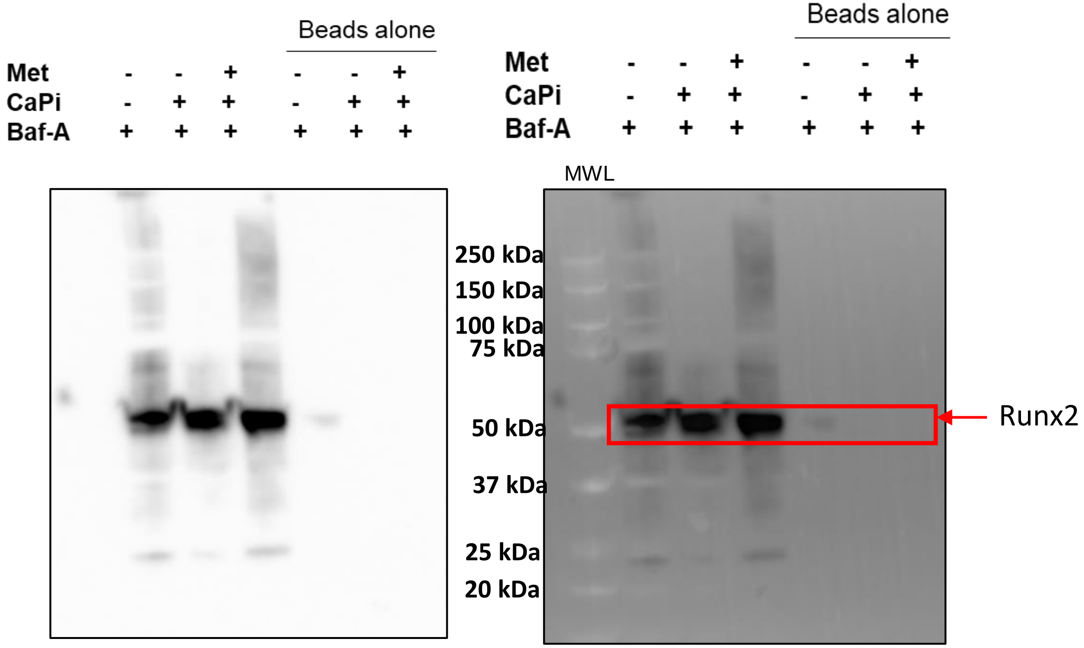

IB: Runx2

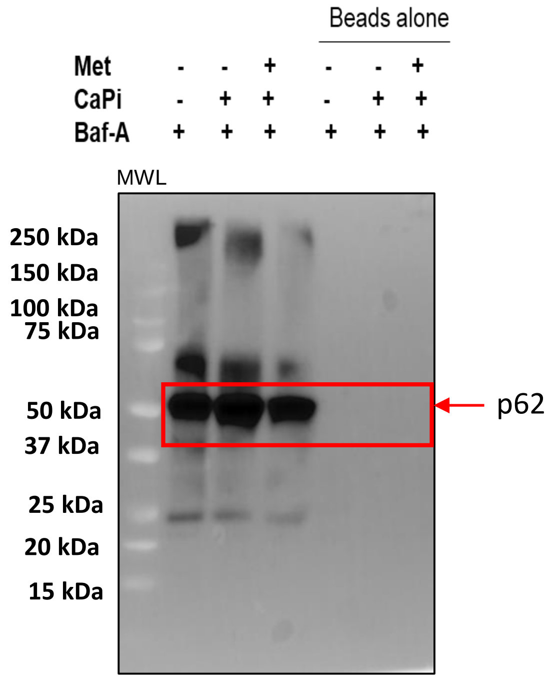

IB: p62

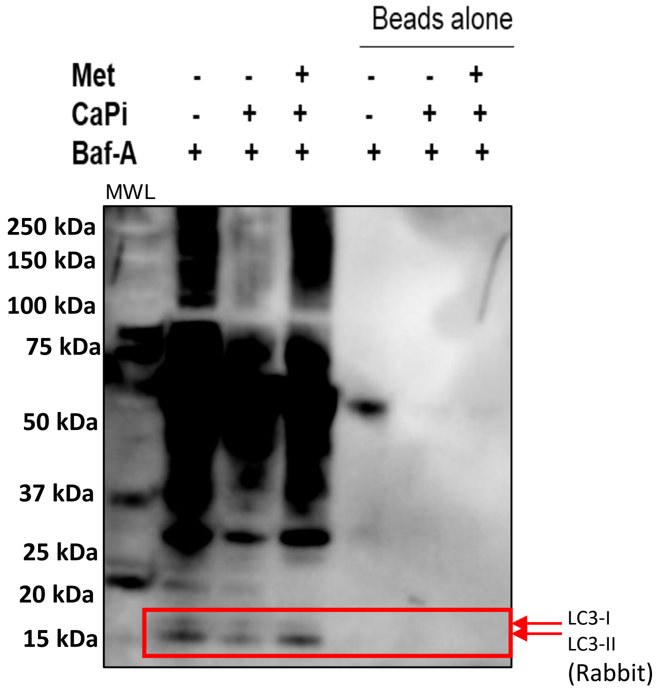

IB: LC3

N=1

Figure 5B IP p62

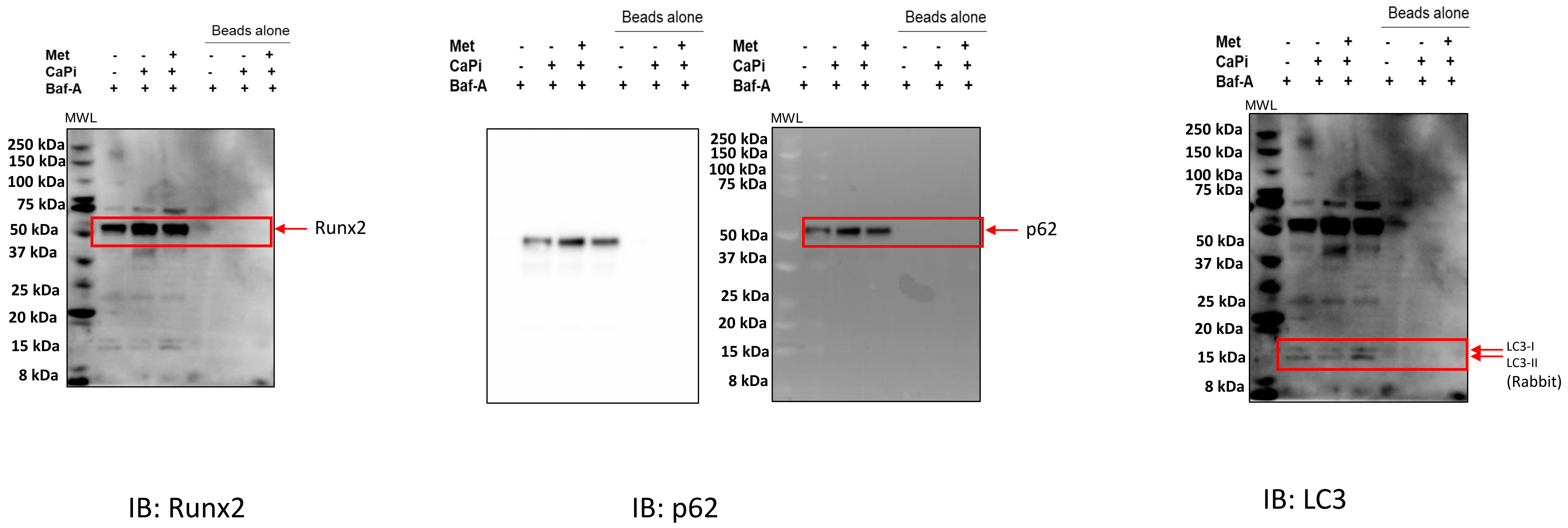

N=1

Figure 5C IP LC3

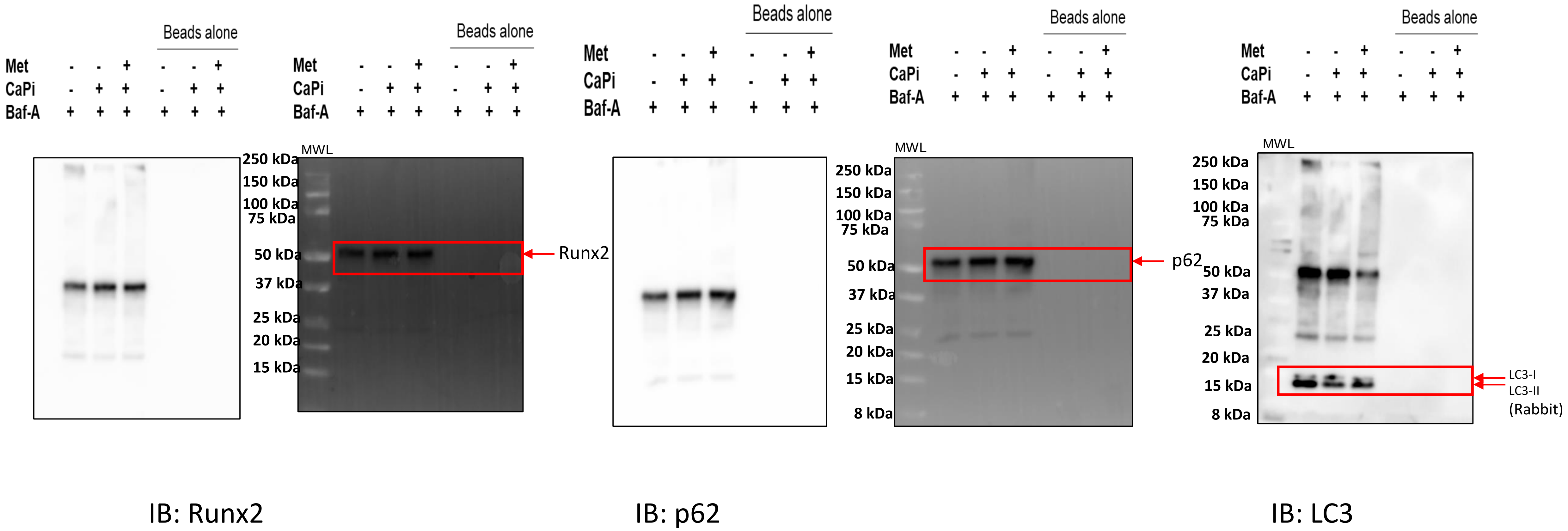

N=1

Figure 5A

IP RUNX2

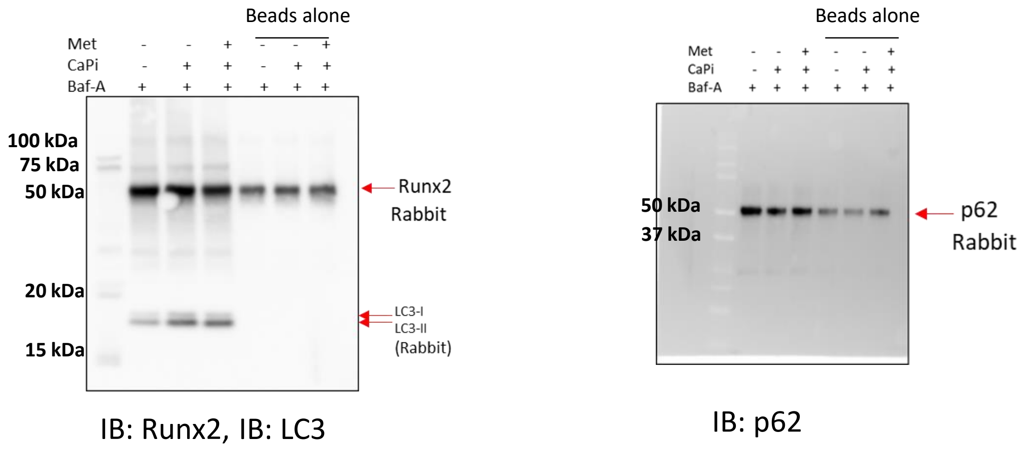

Figure 5B

IP p62

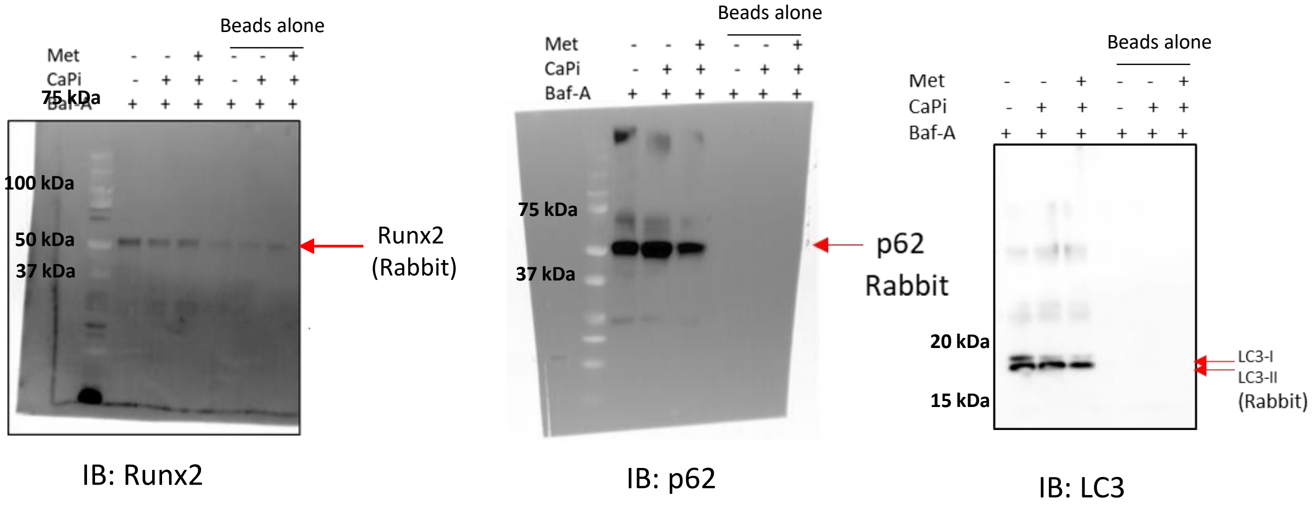

Figure 5C

IP LC3

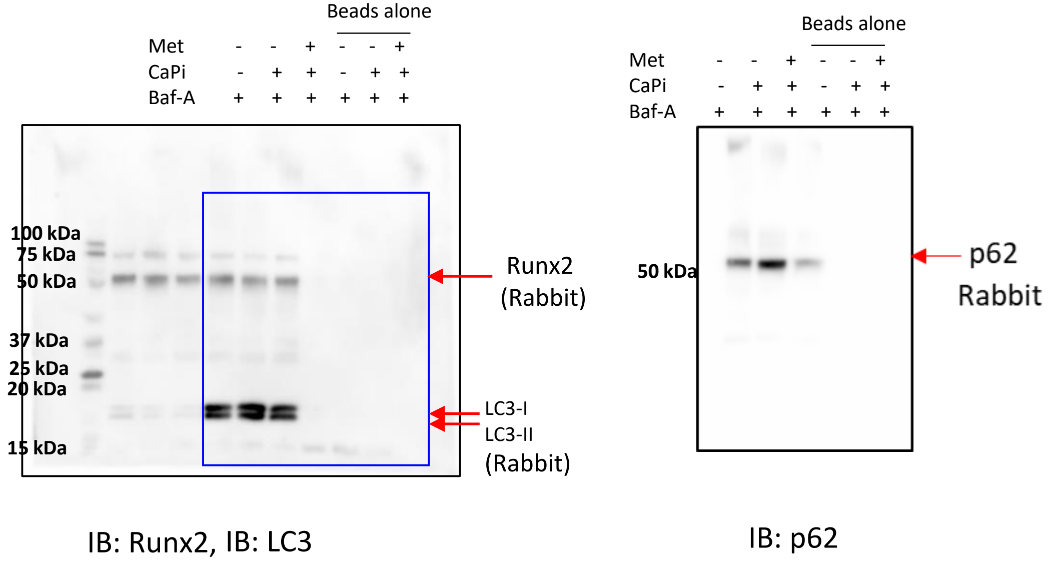

N=2

Figure 5D Input

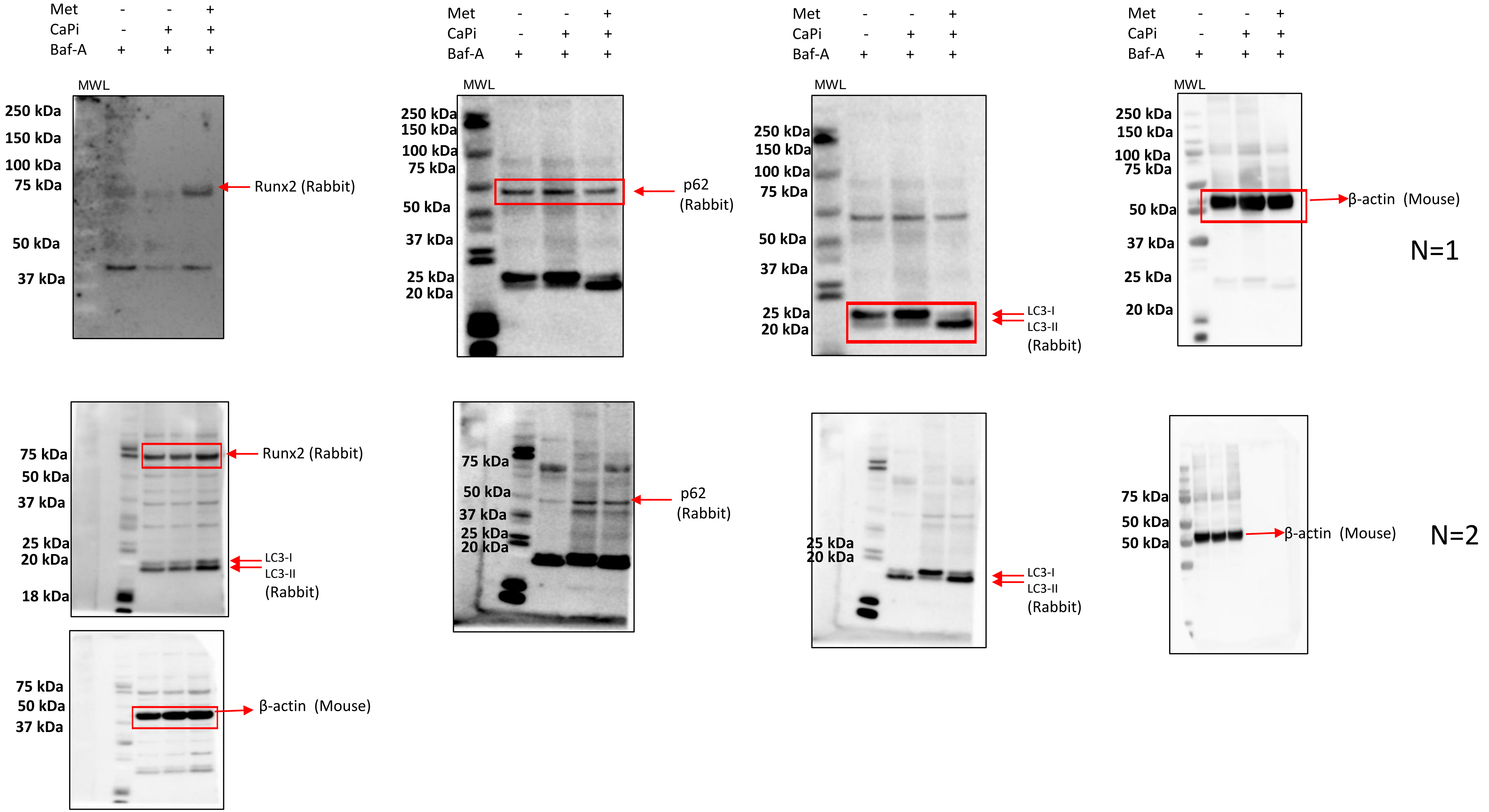

Supplement: Supplementary file 2 — Supplementary Figure 2. [file 41598_2023_47774_MOESM2_ESM.pdf]
